# Elevated Monocyte Chemoattractant Protein-1 as the Independent Risk Factor of Delirium after Cardiac Surgery. A Prospective Cohort Study

**DOI:** 10.3390/jcm10081587

**Published:** 2021-04-09

**Authors:** Jakub Kaźmierski, Piotr Miler, Agnieszka Pawlak, Hanna Jerczyńska, Joanna Woźniak, Emilia Frankowska, Agnieszka Brzezińska, Katarzyna Woźniak, Michał Krejca, Mirosław Wilczyński

**Affiliations:** 1 Department of Old Age Psychiatry and Psychotic Disorders, Medical University of Lodz, 92-216 Lodz, Poland; joannawk24@gmail.com (J.W.); emilia.frankowska@umed.lodz.pl (E.F.); 2 Central Clinical Hospital of Medical University of Lodz, 92-216 Lodz, Poland; drpmiler@gmail.com (P.M.); agnieszkapawlak1502@gmail.com (A.P.); antymonek@hotmail.com (A.B.); 3 CoreLab Central Scientific Laboratory of Medical University of Lodz, Medical University of Lodz, 92-215 Lodz, Poland; hanna.jerczynska@umed.lodz.pl; 4 Department of Cardiac Surgery, Medical University of Lodz, 92-213 Lodz, Poland; kaa.wozniak@gmail.com (K.W.); michal.krejca@umed.lodz.pl (M.K.); miroslaw.wilczynski@umed.lodz.pl (M.W.)

**Keywords:** delirium, cardiac surgery, coronary artery bypass grafting, monocyte chemoattractant protein-1, high-sensitivity C-reactive protein

## Abstract

Background: The pathogenesis of postoperative delirium is largely unknown. The primary objective of this study is to assess whether increased levels of monocyte chemoattractant protein-1 (MCP-1) and high-sensitivity C-reactive protein (hsCRP) are associated with postoperative delirium in patients who have undergone cardiac surgery. The secondary objective is to investigate whether any association between raised inflammatory biomarkers levels and delirium is related to surgical and anesthetic procedures or mediated by pre-existing psychiatric conditions associated with raised pro-inflammatory markers levels. Methods: The patients were screened for cognitive impairment one day preoperatively with the use of the Mini-Mental State Examination Test and the Clock Drawing Test. A diagnosis of major depressive disorder (MDD) and anxiety disorders was established on the basis of Diagnostic and Statistical Manual of Mental Disorders (DSM-5) criteria. Blood samples were collected pre- and postoperatively for hsCRP and chemokine levels. Results: Postoperative delirium developed in 34% (61 of 177) of patients. Both pre- and postoperative hsCRP, and preoperative MCP-1 levels were associated with postoperative delirium in univariate comparisons; *p =* 0.001; *p <* 0.001; *p <* 0.001, respectively. However, according to a multivariable logistic regression analysis, only a raised MCP-1 concentration before surgery was independently associated with postoperative delirium, and related to advancing age, preoperative anxiety disorders and prolonged intubation. Conclusions: The present study suggests that an elevated preoperative MCP-1 concentration is associated with delirium after cardiac surgery. Monitoring of this inflammatory marker may reveal the cardiovascular disease (CVD) patients who are at risk of neuropsychiatric syndromes development.

## 1. Introduction

Cardiac surgery, including coronary artery bypass grafting (CABG), is a lifesaving intervention for severe ischemic heart disease. It substantially improves a patient’s quality of life and prognosis. However, it is often complicated with postoperative delirium.

Delirium affects 4% to 50% of post-operative cardiac surgery patients in intensive care units (ICUs) [1,2,3,4,5] resulting in higher mortality and morbidity rates (sternum instability, reoperation and sepsis), longer stays in the ICU and overall length of admission, as well as greater cost [4,5,6,7,8]. Postoperative delirium may additionally contribute to the development of cognitive decline and institutionalization post-discharge [9].

However, despite its importance, the pathophysiology of postoperative delirium remains poorly understood. One hypothesis suggests that delirium may, in the course of excessive neuro-immune cells stimulation by peripheral inflammation, lead to increased neuroinflammation [10].

MCP-1 is a key chemokine involved in neuroinflammation. In mouse and human brains, MCP-1 and its receptor (CCR2) are primarily expressed by microglia [11], and MCP-1/CCR2 signaling is involved in numerous neuroinflammatory diseases, such as multiple sclerosis [12], stroke [13], and Alzheimer’s disease (AD) [14].

In AD pathology, activated microglia characterized with MCP-1 overexpression constitute the major mechanism of myelin degradation, amyloid deposits and plaques formation, and neuronal loss [15]. In line with this observation is the fact that MCP-1 concentration is increased in the serum and cerebrospinal fluid (CSF) of patients with mild cognitive impairment (MCI) and AD [16].

A relationship between cortisol, postoperative pro-inflammatory cytokine levels and delirium among cardiac surgery patients was reported in our previous studies [17,18]; however, the link between MCP-1 mediated inflammation, its activity and postoperative delirium remains unknown.

Therefore, our primary aim was to investigate the association between raised MCP-1 concentration and postoperative delirium. The secondary aim was to investigate whether any association between increased MCP-1 levels and delirium is related to perioperative and anesthetic procedures or mediated by pre-existing conditions potentially associated with raised chemokine levels, such as affective and/or anxiety disorders, cognitive impairment or aging. In addition, together with MCP-1 concentration, we investigated the high-sensitivity C-reactive protein (hsCRP) levels to determine if the putative association between MCP-1 and delirium is related to systemic inflammation or has more specific character.

## 2. Materials and Methods

### 2.1. Overview

The study was approved by the Ethics Committee of the Medical University of Lodz, Poland and was performed in accordance with the ethical standards of the Declaration of Helsinki. The study was conducted in the 14-bed cardiac surgical intensive care unit (ICU) of a university teaching hospital (Central Clinical Hospital, Medical University of Lodz, Poland) between April 2017 and November 2019. The subjects signed their informed consent the day before their operation. The inclusion criteria were consecutive adult patients scheduled for CABG surgery or CABG surgery with cardiac valve repair or replacement (CVR). Patients who underwent CABG were eligible both in the case of on-pump and off-pump surgery; however, the impact of the cardiopulmonary bypass (CPB) on the risk of postoperative delirium was controlled in the statistical analysis. The exclusion criteria were as follows: concomitant surgery other than CABG or CABG with CVR; preoperative delirium; active alcohol or other substances addiction (abstinence period shorter than 3 months); illiteracy; patients on dietary supplements; and pronounced hearing and/or visual impairment.

### 2.2. Preoperative Psychiatric and Psychological Procedures

The study population was examined by a psychiatrist the day prior to the scheduled operation using the Mini-Mental State Examination (MMSE) and Clock Drawing Test (CDT) to assess the global cognitive status of the participants [19,20]. A diagnosis of MDD and anxiety disorders (generalized anxiety disorder, panic disorder, mixed anxiety disorder) was established by the psychiatrist on the basis of Diagnostic and Statistical Manual of Mental Disorders (DSM-5) criteria [21].

### 2.3. Anesthesia and Surgery

For premedication, midazolam (7.5 mg p.o.) a half-hour before the surgery was used. Before induction of anesthesia in all patients, routine monitoring was installed: electrocardiography, invasive arterial blood pressure monitoring, central venous pressure monitoring, cerebral oxygen saturation, peripheral oxygen saturation and urine output. A standard anesthesia technique was performed in all patients (induction: fentanyl 5–10 mcg/kg, propofol 1–2.5 mg/kg and rocuronium 0.6–1.0 mg/kg). Medication during maintenance was as follows: fentanyl in continuous intravenous infusion in doses of 2–10 mcg/kg/h, propofol 3–10 mg/kg/h, and interrupted doses of rocuronium. Ventilation was provided with breathing a mixture of FiO_2_ 0.5 and air to maintain end-tidal CO_2_ at 35–45 mmHg. From surgical incision to cardiopulmonary bypass connection, sevoflurane 0.5–2 vol% was used. In case of hypotension, ephedrine in boluses or norepinephrine in continues infusion was used to counteract profound vasodilation to maintain mean arterial pressure above 55 mmHg. After the operation, patients were transferred to the ICU and were placed on mechanical ventilation. Until extubation, they were sedated with morphine in a continuous infusion of 1–2 mg per hour and propofol perfusion at a rate of 1–2 mg/kg/h. The acceptable levels of arterial blood gases and oxygen sat at >92% and stabilization of hemodynamic parameters were the criteria for extubation, which, in uncomplicated cases, took place 4–8 h after the operation. Patients who underwent CABG or CABG with concomitant valve surgery were operated through median sternotomy and on CPB under normothermia. The anterograde DelNido cardioplegia was used for cardiac protection during the operation. In some cases, patients who underwent coronary revascularization were operated without CPB (off-pump CABG-OPCAB) on a beating heart, either through the median sternotomy or through left-sided mini-thoracotomy.

### 2.4. Measurement of Serum MCP-1 and hsCRP Concentration

The venous blood samples were taken the day prior to the surgery (baseline measurement) and on the day after intervention, between the hours of 07:00 and 09:00 a.m. The blood samples were centrifuged at 7669× *g* for 10 min, were frozen at −80 °C and stored as a single aliquot until the biochemical parameters were determined. The MCP-1 and hsCRP levels were measured in serum samples using commercially available ELISA kits (R&D, USA for MCP-1, and DRG International, USA for hsCRP). Samples were run in duplicate. The intra-assay CV mean was 2.8% (0–8.8%) and 1.6% (0–7.8%) for hsCRP and MCP-1, respectively. The hsCRP analysis samples taken prior to the surgery were diluted 10-fold, and those after the surgery, 500-fold. In MCP-1, all samples were diluted 2-fold. Protocols were performed according to the manufacturer’s instructions.

The ELISA results were analyzed with WorkOut 2.5 Software (WorkOut™ is a data analysis software provided with Victor X4 multilabel plate reader by Perkin Elmer) and the mean concentration of protein per ml was determined by referring to the four parameter logistic (4-PL) curve. Standard curves were run on every ELISA plate. Inter-assay was 4.1% and 2.1% for hsCRP and MCP-1, respectively.

The tests were conducted by investigators that were blinded to the clinical data.

### 2.5. Delirium Diagnosis

Following surgical interventions, the Confusion Assessment Method for the Intensive Care Unit (CAM for ICU) and the Memorial Delirium Assessment Scale (MDAS), with the cut-off score of 10, were used to diagnose delirium [22,23]. Each individual was assessed by a psychiatrist once a day within the first 5 days after surgery. Before each administration of the CAM for the ICU Unit, the level of sedation/arousal was assessed using the Richmond Agitation Sedation Scale (RASS) [24]. If the patient was deeply sedated or was unarousable, which corresponds to −4 or −5 on the RASS, the evaluation was stopped and repeated later. If the RASS was above −4 (−3 through +4), the assessment with the CAM for ICU was administered. If there was an inconsistency between the diagnostic tools regarding the delirium diagnosis, the final consensus was established within the study team physicians collecting information from all available sources.

### 2.6. Statistical Analysis

Quantitative variables are expressed as medians and interquartile ranges (IQRs), and means and ranges. For categorical variables, the number of observations (n) and fraction (%) were calculated.

Normality was tested using the Shapiro−Wilk’s test for normality. Differences between two independent samples for continuous data were analyzed using the Mann–Whitney U test (since the distributions of variables were different from normal). The effect size for continuous variables was measured with the rank-biserial correlation coefficient.

For categorical variables, the statistical analysis was based on the chi-squared test or Fisher’s exact test. Cramer’s V coefficient was calculated to assess the effect size for categorical variables. Spearman’s rank correlation coefficients were calculated to assess the correlation between two quantitative variables. A nonparametric analysis of variance of the aligned rank transformed data (ART) was used to compare the levels of markers in different groups of patients (taking into account two qualitative factors). The minimum study sample size was calculated using the power analysis, estimating the expected effects from our previous studies and assuming an alpha level of 0.10 and a power of 80% (minimum sample size for each group is 37 patients). Initially, baseline and perioperative variables were evaluated for univariate association with postoperative delirium. For quantitative variables (preoperative and postoperative antioxidant activity) significantly associated with the occurrence of delirium, receiver operating characteristic (ROC) curves were drawn (Area Under Curve with Standard Error was given) and optimal decision thresholds (based on the Youden’s index value) were found. The sensitivity, specificity, positive predictive value and negative predictive value were calculated. Odds ratios with 95% confidence intervals were also presented. Factors significant in univariate comparisons (*p* < 0.10) were included in a forward stepwise logistic regression model to identify the set of the independent risk factors for delirium. The results were considered significant for *p* < 0.05. All of the calculations were performed using STATISTICA (version 13.3, 2017; StatSoft Inc., Tulsa, OK, USA) and the R-project (the rcompanion package).

## 3. Results

### 3.1. Basic Findings

After approval by the Ethics Committee of the Medical University of Lodz, 294 patients scheduled for cardiac surgery were assessed for eligibility for participation in the study. Of these, 70 subjects did not meet the inclusion criteria since they underwent surgery different than CABG/CABG with valve surgery (*n =* 58) or refused to participate (*n =* 12). Of the 224 patients who signed their informed consent and were enrolled, four patients were lost to follow-up since they died before the observational period was completed, and 43 individuals had incomplete study data (these patients were not included in the analysis due to failure during samples collection or inappropriate samples collection (coagulation), *n =* 28; patients with incomplete postoperative delirium assessment, *n =* 15). The remaining 177 patients were entered into the analysis.

The median age of the population was 67 years (IQR: 63–71) and did not differ between women and men (69 vs. 66.6 respectively, *p =* 0.24); 138 (78%) of the participants were men. Cognitive impairment (MMSE score < 25) was diagnosed in 15 (8.5%) patients, whereas MDD and anxiety disorders were diagnosed in 33 (18.6%) and 14 (7.9%) individuals, respectively. The prevalence of anxiety disorders was higher among women (17.95%, *n =* 7) compared to men (5.07%, *n =* 7; *p =* 0.01). There were no significant differences in the prevalence of depression between women and men (23.08%, *n =* 33 vs. 17.39%, *n =* 24, respectively; *p =* 0.42) and the median MMSE score (28 vs. 28, respectively; *p =* 0.85). None of the participants suffered from a systemic inflammatory disease. None of the patients had preoperative delirium. There were 63 patients diagnosed with delirium according to the CAM for ICU, and 58 patients diagnosed with delirium according to the MDAS. After the study team discussion and the patients’ data analysis, postoperative delirium was finally diagnosed in 61 subjects (34%). The median preoperative and postoperative MCP-1 concentration in the whole population was 385.48 ng/mL (IQR: 317.6–498.7) and 520.0 ng/mL (IQR: 365.7–748.2), respectively. Descriptive statistics for the delirium and non-delirium group, as well as the results of the univariate analysis are presented in Table 1, Table 2 and Table 3.

### 3.2. Variables Associated with Delirium according to Univariate and Multivariable Analysis

1. According to univariate analysis, the individuals with raised pre-, and postoperative hsCRP, and preoperative MCP-1 levels were at higher risk of postoperative delirium compared to patients with lower hsCRP and MCP-1 concentrations (Table 2). Interestingly, after controlling for variables significant in univariate comparisons, only individuals with an MCP-1 level increased before surgery remained at increased risk of postoperative delirium development (Table 4). Other factors independently associated with delirium included: age, gender female, MDD diagnosis, peripheral vascular disease diagnosis, and the presence of extracorporeal circulation (Table 4).
2. The most optimal cut-off for preoperative MCP-1 concentration in predicting the development of delirium was 371.81 ng/mL with sensitivity of 77.0% and specificity of 58.6%.

### 3.3. Correlations between MCP-1 Concentration, Demographic and Perioperative Variables

1. Patients aged 65 and more who developed delirium after surgery had significantly higher baseline MCP-1 concentrations compared to younger, non-delirious subjects (466.66 ng/mL; IQR: 371.81–554.67 vs. 326.96 ng/mL; IQR: 263.01–408.31, *p =* 0.008).
2. Preoperative MCP-1 level was also increased in individuals with a diagnosis of anxiety disorders who developed postoperative delirium compared to patients without anxiety disorders and without postoperative delirium (546.65 ng/mL; IQR: 450.45–598.58 vs. 352.44 ng/mL; IQR: 288.13–448.81, *p =* 0.05).
3. Moreover, a nonparametric analysis of variance of aligned rank transformed data (ART) showed significant interaction for gender female and delirium with regard to the postoperative MCP-1 concentration (partial eta-squared = 0.023; *p =* 0.046). According to the post hoc pairwise comparisons, median postoperative MCP-1 concentration was increased among women who developed delirium compared to non-delirium women (678.46 ng/mL; IQR: 408.64–816.40 vs. 366.54 ng/mL; IQR: 273.18–520.03, *p =* 0.002), whereas there were no MCP-1 differences between men with and without delirium (*p =* 0.207).
4. Furthermore, positive correlations between more advanced age, prolonged intubation time, and MCP-1 concentrations were observed (Table 5).
5. According to the ART analysis, there were no significant differences in pre- and postoperative MCP-1 levels between patients with depression (*p =* 0.33; *p =* 0.85, respectively), cognitive impairment (*p =* 025; *p =* 022, respectively), undergoing CABG plus CVR surgery (*p =* 0.90 for postoperative MCP-1), and surgery with ECC (*p =* 0.38 for postoperative MCP-1) compared to patients without depression, without cognitive impairment, and CABG only and on-pump surgery. Furthermore, there were no significant correlations between MCP-1 concentrations and MMSE score, CDT score, surgery time and aortic cross-clamping, as well as pre- and postoperative hsCRP levels (Table 5).

## 4. Discussion

This is a prospective study in which the association between perioperative hsCRP and MCP-1 concentrations and postoperative delirium was elucidated. According to the multivariable regression model, patients with a raised concentration of MCP-1 measured the day before the operation were significantly more likely to develop delirium. This association was independent of age, gender, psychiatric and physical comorbidity and CPB presence.

CABG surgery prolongs life and improves the physical condition of patients with severe CAD; however, these benefits are accompanied by postoperative mortality, stroke, atrial fibrillation, and neurocognitive dysfunction, including delirium and dementia [5,8,9]. Cardiac surgery activates endocrine and immune changes classified as the “stress response” [25]. Perioperative inflammatory interactions and pathways include generation and activation of complement, cytokines, neutrophils, macrophages, thrombin and mast cells [26]. However, an excessive inflammatory response can disrupt the balance between health and disease, and lead to self-attack of the host cells, resulting in organ injury and neuropsychiatric-adverse postoperative outcomes [27]. Inflammation has been linked to delirium in the population of hospitalized critically ill patients [28,29]. Moreover, in studies conducted among patients presented for hip surgery, differences between individuals with and without delirium were observed in plasma and cerebrospinal fluid IL-6 and IL-8 levels [30,31]. Other study described median preoperative IL-6 levels in the patients with postoperative delirium vs. the non-delirium group as 9 pgmL-1 vs. 3.4 pgmL-1 [32]. It should be noted, however, that some of above cohorts included both patients who developed delirium prior and after surgery. In addition, the aforementioned studies used a case-control design or univariate analysis only.

Our previous prospective study conducted among cardiac surgery patients revealed that raised levels of IL-2 and TNF-α measured in the postoperative period are associated with the development of delirium among CABG surgery patients [17]. This higher concentration of pro-inflammatory cytokines was additionally associated with a longer duration of CPB (IL-2 only) and related to advancing age but importantly, not to a preoperative diagnosis of MDD. It should be noted, however, that in this study, the preoperative levels of cytokines were not investigated. Therefore, a causative effect of preoperative cytokine activity on the development of delirium was not determined.

Monocyte chemotactic protein-1 (MCP-1; chemokine C-C ligand-2) is a member of the chemokine family. Monocytes express CCR-2 (the receptor for MCP-1), and MCP-1 itself regulates trafficking of monocytes from the bone marrow to inflamed tissue in response to inflammatory signals [33]. In experimental studies, MCP-1 expression was upregulated immediately after ischemia reperfusion injury, and MCP-1 inhibition was associated with lower cardiac fibrosis [34,35]. Human studies revealed MCP-1 elevation immediately after cardiac surgery [36,37], and MCP-1 overexpression was associated with acute kidney injury and, as a consequence, increased risk of dying [38]. MCP-1 concentration is also increased in other conditions including lupus nephritis, diabetic nephropathy, infection, allergic reactions, bone remodeling, atherosclerosis, and inflammatory bowel disease [39]. Despite the role of MCP-1 in the inflammatory response and various pathologies, the association between this chemokine and post-surgery neuropsychiatric complications has not been investigated.

To our knowledge, this is the first study to indicate that a relationship between increased MCP-1 levels and delirium after cardiac surgery exists. A univariate analysis revealed that preoperative elevation of hsCRP and MCP-1 and postoperative increase in hsCRP are associated with increased risk of delirium development; however, only preoperative MCP-1 concentration was significant in the multivariable logistic regression model. Interestingly, the postoperative MCP-1 concentration was higher among female patients with delirium compared to women who did not develop delirium, and this relationship was not observed among males. As gender female was an independent predictor of postoperative delirium according to the current analysis, the higher post-surgery MCP-1 peak seen in female patients may underpin an increased risk of delirium development in this group. Previous studies revealed that MCP-1 levels measured in postmenopausal women are associated with subclinical atherosclerosis disease severity [40]. Moreover, MCP-1 levels are positively correlated with intermuscular adipose tissue whose content is higher among women and constitutes a major risk factor for insulin resistance, diabetes and all-cause mortality [41]. Of note, gender female was not reported to increase the risk of postoperative delirium in the previous analyses. In some studies conducted in non-cardiac cohorts, male sex was associated with postoperative neuropsychiatric complications [42]. This difference may be due to the specificity of the cardiac surgery population compared to patients scheduled for orthopedic or other non-cardiac operations (higher psychiatric comorbidity, gender-related differences in delirium biomarkers associated with atherosclerosis) [18]. In the present study, women more frequently suffered from anxiety disorders and depression (insignificant difference) compared to men. Moreover, subjects with active alcohol addiction were excluded from the analysis. As alcohol addiction is more prevalent among men and may be complicated with alcohol withdrawal delirium, this variable could be responsible for some of the delirium cases seen among men in studies which did not specify alcohol dependence as an exclusion criterion.

The mechanism responsible for elevated preoperative levels of MCP-1 is unknown; nevertheless, the present analysis suggests that it may be related to more advanced age and the presence of anxiety disorders, whose prevalence is high among cardiac patients. Studies report the relationship between inflammatory factors, aging and anxiety disorders [43,44]. In the present study, elderly patients and individuals with a diagnosis of anxiety disorders had higher preoperative MCP-1 levels compared to younger patients and subjects without anxiety disorder diagnoses. The above associations suggest that an elevated preoperative MCP-1 level is related both to demographic and psychiatric status, and independently increases the risk of post-surgery delirium development. Interestingly, although both pre- and postoperative hsCRP were significantly increased in delirious patients in statistical comparisons between the groups, this marker did not enter the final multivariable model. Moreover, there were no significant correlations between MCP-1 and hsCRP concentration. These findings suggest that in the cardiac surgery population, elevated CRP levels have weaker prognostic relevance compared to other more specific factors. Moreover, the lack of correlation between the hsCRP and MCP-1 values may result from different pathophysiological mechanisms of the two parameters. The vast majority of CRP is synthesized by hepatocytes in response to the stimulation of such cytokines as IL-6 and TNF-α [45]. However, MCP-1 may be secreted by neutrophils and expressed by endothelial cells of cardiac or neural tissue vessels, even in the absence of stimulation by MCP-1 specific cytokines (IL-1) and blood components [46,47]. The predictive value of MCP-1 instead of CRP was also reported in studies devoted to biomarkers of community-acquired pneumonia [47], hemorrhagic stroke and cardiovascular disease (CVD) mortality [48,49]. In the latter study, hsCRP was not related to CVD (*p =* 0.22), and MCP-1 showed a trend significantly associated with CVD mortality in an age- and sex-adjusted multivariable analysis (*p =* 0.059). No correlation between plasma MCP-1 and hsCRP was detected.

### Study Limitations

The study group is rather heterogenous since both patients with CABG and valve surgery, as well as on-pump and off-pump surgery, were included. On the other hand, the aim of the current study was to determine the mechanisms underpinning postoperative delirium development and their putative associations with such factors as psychiatric and physical comorbidity, type of surgery or the presence of extracorporeal circulation. Moreover, different surgery types and techniques were included into the statistical analysis as potential confounders. The potential bias of the study results associated with the patients with incomplete study data who were excluded from the analysis should be noted (43 subjects). These were accidental patients excluded from the analysis throughout the whole study period; thus, any methodical error should not occur.

## 5. Conclusions

Patients with raised preoperative MCP-1 concentrations are more likely to develop delirium after cardiac surgery. Preoperatively raised MCP-1 may be associated with more advanced age, a diagnosis of anxiety disorders and prolonged intubation. The postoperative peak of MCP-1 concentration seen among women may underly the association between gender female and post-surgery delirium. Given the association between MCP-1 and delirium, MCP-1 receptor blocker development may be considered in future studies devoted to postoperative delirium prophylaxis and treatment.

## Figures and Tables

**Table 1:** Preoperative variables related to demography and physical condition of patients analyzed in a univariate analysis.

Variable	Non-Delirious ^a^ (*n =* 116)	Delirious ^a^ (*n =* 61)	Effect Size ^b^	*p*
Age (years)	66 (61–69)	70 (66–72)	−0.340	<0.001 *
Gender female	15 (13.0%)	24 (39.0%)	0.303	<0.001 *
Peripheral vascular disease	13 (11.2%)	17 (27.9%)	0.211	0.005 *
Arterial hypertension	89 (76.7%)	56 (91.8%)	0.186	0.013 *
NYHA	2 (2–2)	2 (2–3)	−0.175	0.029 *
Atrial fibrillation	10 (8.6%)	12 (19.7%)	0.159	0.034 *
Diabetes	35 (30.0%)	26 (42.6%)	0.125	0.098 *
Creatinine concentration (mcmol/L)	83.7 (75.4–98.3)	88 (68.1–104.8)	−0.028	0.758
Anemia ^c^	16 (13.8%)	11 (18.0%)	0.056	0.456
Cerebrovascular disease	12 (10.3%)	9 (14.7%)	0.065	0.464
COPD	6 (5%)	5 (8.25)	0.060	0.516
CCS	2 (2–3)	2 (2–3)	0.115	0.503

CCS: Canadian Cardiovascular Society degree; COPD: Chronic obstructive pulmonary disease; NYHA: New York Heart Association grade. ^a^ For continuous variables, the medians and interquartile ranges (IQRs) are given; for categorical variables, the number of observations (*n*) and fraction (%) were calculated; ^b^ for continuous variables, rank-biserial correlation coefficient was calculated; for categorical variables, Cramer’s V coefficient was given; ^c^ hemoglobin concentration <10 mg/dL; * indicates significance (*p* < 0.1).

**Table 2:** Monocyte chemoattractant protein-1 and high-sensitivity C-reactive protein levels, and preoperative variables related to the mental condition of participants assessed in a univariate analysis.

Variable	Non-Delirious ^a^ (*n =* 116)	Delirious ^a^ (*n =* 61)	Effect Size ^b^	*p*
Depression	9 (7.8%)	24 (39.0%)	0.385	<0.001 *
Anxiety disorders	5 (4.3%)	9 (14.7%)	0.184	0.02 *
Alcohol addiction ^c^	8 (6.9%)	5 (8.2%)	0.024	0.768
MMSE score	28 (26–29)	28 (26–29)	0.130	0.149
CDT score	7 (5–7)	6 (5–7)	0.066	0.458
Preoperative MCP-1 (ng/mL)	353.4 (290.7–446.0) 400.3 (114.6–1416.4) ^d^	458.4 (374.1–554.7) 483.5 (174.33–987.6) ^d^	−0.404	<0.001 *
Postoperative MCP-1 (ng/mL)	486.9 (359.8–712.1) 671.2 (202.0–2069.3) ^d^	598.9 (386.1–765.5) 712.5 (154.0–10688.1) ^d^	−0.129	0.16
Preoperative hsCRP (mcg/mL)	2.97 (0.94–7.57) 9.92 (0.04–151.5) ^d^	7.6 (2.2–12.0) 10.7 (0.08–102.7) ^d^	−0.302	0.001 *
Postoperative hsCRP (mcg/mL)	151.6 (93.9–207.2) 177.3 (9.5–2116.1) ^d^	212.88 (124.6–277.6) 266 (30.4–4009.6) ^d^	−0.322	<0.001 *

CDT: Clock Drawing Test; hsCRP: High-sensitivity C-reactive protein; MMSE: Mini-Mental State Examination; MCP-1: Monocyte chemoattractant protein-1. ^a^ For continuous variables, the medians and interquartile ranges (IQRs) are given; for categorical variables, the number of observations (*n*) and fraction (%) were calculated; ^b^ for continuous variables rank-biserial correlation coefficient was calculated; for categorical variables, Cramer’s V coefficient was presented; ^c^ only patients with at least 3 months abstinence were included; ^d^ for more detailed presentation of the data, the means and ranges were also given; * indicates significance (*p <* 0.1).

**Table 3:** Perioperative variables analyzed in univariate analysis.

Variable	Non-Delirious ^a^ (*n =* 116)	Delirious ^a^ (*n =* 61)	Effect Size ^b^	*p*
CABG with valve surgery	8 (6.9%)	9 (14.75%)	0.127	0.092 *
ECC	81 (69.8%)	52 (85%)	0.170	0.024 *
Hyperthermia ^d^	9 (7.8%)	10 (16.4%)	0.133	0.078 *
Aortic cross-clamping ^c^ (min.)	40 (30–55)	43 (30–70)	−0.114	0.270
Duration of surgery (h)	4.0 (3–4.5)	4.0 (4–4.5)	−0.085	0.350
Circulatory support ^c^	2 (1.70%)	1 (1.6%)	0.003	0.97
Corticosteroids use ^c^	0 (0%)	1 (1.6%)	0.104	0.345
pCO_2_ ≥ 45 ^d^ (mmHg)	24 (20.7%)	18 (29.5%)	0.099	0.19
pO_2_ ≤ 60 ^d^ (mmHg)	18 (15.5%)	13 (21.3%)	0.072	0.33

CABG: Coronary artery bypass graft surgery; ECC: Extracorporeal circulation. ^a^ For continuous variables, the medians and interquartile ranges (IQRs) are given; for categorical variables, the number of observations (*n*) and fraction (%) were calculated; ^b^ for continuous variables rank-biserial correlation coefficient was calculated; for categorical variables, Cramer’s V coefficient was given; ^c^ intraoperative variables; ^d^ postoperative variables; * indicates significance (*p <* 0.1).

**Table 4:** Factors independently associated with delirium after cardiac surgery revealed in multivariable stepwise logistic regression analysis ^a^.

Variables	Coefficient	Standard Error	OR (95% CI)	*p*
MCP-1 ^b^	0.002	0.001	1.002 (1.000–1.004)	0.050
Depression ^b^	2.360	0.518	10.59 (3.835–29.238)	0.000
Gender female	1.779	0.465	5.992 (2.380–14.735)	0.000
Age	0.085	0.033	1.089 (1.021–1.161)	0.010
ECC	1.253	0.539	3.5 (1.217–10.072)	0.020
Peripheral vascular disease ^b^	1.248	0.503	3.483 (1.300–9.331)	0.013
Preoperative hsCRP ^c^	0.015	0.009	1.015 (0.998–1.032)	0.094
Postoperative hsCRP ^c^	0.001	0.001	1.001 (0.999–1.003)	0.340
Constant	−9.435	2.336	-	0.000

ECC: Extracorporeal circulation; hsCRP: High-sensitivity C-reactive protein; MCP-1: Monocyte chemoattractant protein-1. ^a^ The regression model is statistically significant: χ^2^ = 42.023, df = 6, *p <* 0.001; Hosmer–Lemeshow test: χ^2^ = 4.668, *p =* 0.792; Nagelkerke R2 = 0.447; ^b^ preoperative variables; ^c^ insignificant variables.

**Table 5:** The correlations between pre- and postoperative MCP-1 levels and demographic, psychiatric and perioperative variables.

Variables	Spearman’s Rank Correlation	*p*
Preop-MCP-1 and age	0.192	0.01 *
Postop-MCP-1 and age	0.059	0.43
Preop-MCP-1 and MMSE score	−0.029	0.69
Postop-MCP-1 and MMSE score	−0.080	0.28
Preop-MCP-1 and CDT score	−0.062	0.41
Postop-MCP-1 and CDT score	−0.092	0.22
Postop-MCP-1 and surgery time	0.039	0.60
Postop-MCP-1 and aortic cross-clamping time	0.124	0.15
Preop-MCP-1 and intubation time	0.279	0.0002 *
Postop-MCP-1 and intubation time	0.148	0.048 *
Preop-MCP-1 and preop-hsCRP	0.11	0.13
Postop-MCP-1 and postop-hsCRP	0.03	0.66

CDT: Clock Drawing Test; hsCRP: High-sensitivity C-reactive protein; MMSE: Mini-Mental State Examination; MCP-1: Monocyte chemoattractant protein-1; * indicates significant correlation (*p <* 0.05).

## Data Availability

The datasets used and/or analyzed during the current study are available from the corresponding author on reasonable request.
